# Resection of a pineal region papillary tumor using robotic exoscope: improved visualization and ergonomics for deep seeded tumor

**DOI:** 10.3171/2021.4.FOCVID2127

**Published:** 2021-07-01

**Authors:** Wei X. Huff, Andrew J. Witten, Mitesh V. Shah

**Affiliations:** Department of Neurological Surgery, Indiana University School of Medicine, Indianapolis, Indiana

**Keywords:** pineal tumor, exoscope, pineal region microsurgery, infratentorial, supracerebellar approach

## Abstract

Surgery for pineal region tumors is technically challenging due to their deep location and close proximity to critical deep venous structures, midbrain, and thalamus. A high-definition video exoscope was recently proposed as an alternative to the operating microscope. The authors illustrate a case of the midline supracerebellar infratentorial approach to resect a pineal region tumor using the Modus V exoscope and demonstrate the improved visualization of critical structures in this deep location. Additionally, the marked improvement in surgeon comfort suggests that this system may have significant advantages over traditional microscope-based surgery for tumors of the pineal region.

The video can be found here: https://stream.cadmore.media/r10.3171/2021.4.FOCVID2127.

## Transcript

We are presenting a video of resection of a pineal region papillary tumor using digital robotic exoscope Modus V [Synaptive Medical]. We would like to demonstrate the advantages of using the exoscope to achieve excellent visualization for deep surgical corridor and improved surgeon’s comfort.

### 0:46 Patient Presentation

This is a 34-year-old right-handed female who presented to the outside hospital with 3-month history of progressive headaches, balance issue, memory difficulties, and visual changes including blurry vision and “tired eyelids.” She needed to blink a lot to focus. Her physical exam revealed mild defects with accommodation and convergence. Her imaging demonstrated a pineal region heterogeneously enhancing lesion with obstructive hydrocephalus. This lesion measured 2.7 × 2.2 × 2.2 cm with areas of calcification and hemorrhage present. She underwent endoscopic third ventriculostomy (ETV) and endoscopic biopsy of the lesion. Her CSF markers were negative for germ cell tumor. Her pathology results were consistent with a papillary tumor. Our tumor board recommended surgical resection. Of note, following ETV, the majority of her symptoms improved. She remained neurologically normal except for mild convergence difficulty.

### 2:03 Preoperative MRI

Here is her preoperative MRI with gadolinium contrast, which shows the heterogeneously enhancing lesion in the pineal region. Of particular interest, the internal cerebral veins are superior to the tumor capsule, and the basal veins of Rosenthal are draped on the supralateral aspects of the tumor capsule.

### 2:28 Microsurgial Approach

Traditionally, microsurgical approaches for the pineal/posterior third ventricular region include midline supracerebellar infratentorial, paramedian supracerebellar infratentorial, and occipital interhemispheric transtentorial approaches.^1–3^ We chose the midline supracerebellar infratentorial approach in the sitting position to take advantage of the gravity-assisted cerebellar retraction. This creates a wide operating corridor to the pineal region. In addition, midline positioning is less likely to cause disorientation with the deep structures. However, one should keep in mind the drawbacks with this approach, which include higher risk of air embolism and premature surgeon fatigue.^3^ The latter is due to the long focal distance of microscope and the need to operate with the arms outstretched. This awkward positioning results in surgeon fatigue and is less than optimal comfort for dissection of delicate structures.

### 3:39 Robotic System

We took advantage of a high-definition robotic exoscope (Modus V) system to ease the surgeon’s positional fatigue and provide superb image quality and illumination to surgical area.^2,4,5^ This demonstrates our OR setup. The patient is in the sitting position with a precordial Doppler in place. The exoscope is set up on the side of the surgeon so that surgeon’s arms are not outstretched and surgeon’s hands can comfortably be resting on the shoulder of the patient for stabilization and comfort. The monitor is ergonomically positioned to alleviate stress on the surgeon’s arms and neck. As we will demonstrate in the surgical video, the exoscope provides excellent depth of field and field of view. One of the key robotic features is the preregistered suction providing automatic focus as the surgeon works in and out of the deep field.

### 4:47 Surgical Procedure

After a standard midline suboccipital craniotomy, we exposed the transverse sinus and torcula to facilitate the superior retraction of the tentorium.

**5:00** The arachnoid adhesions attaching the cerebellum to the tentorium were sharply dissected. The cerebellum falls nicely with gravity, and this provides us a wide surgical corridor.

**5:14** We then identified and isolated the precentral cerebellar vein and divided it. Now the tumor capsule is visible in front of us.

**5:30** We started working in a circumferential fashion to define the plane between the tumor capsule and the normal brain in the surrounding area.

**5:45** We started from the right side of the capsule and worked superiorly to detach the tumor from the roof of the third ventricle.

**6:11** Here you can see the CSF egress from the third ventricle.

**6:32** We then worked inferiorly to release the tumor from the posterior commissure. There were more adhesions inferiorly and to the left side of the tumor.

**7:19** We continue to dissect the tumor from the underlying surrounding brain.

**7:41** After the majority of the tumor was defined, we debulked tumor to further mobilize the capsule.

**8:06** Now, you can see a large portion of the tumor has been removed. The navigated suction tip can be used to robotically align the exoscope and focus automatically.

**8:29** We have now removed the remaining portion of the tumor.

### 8:41 Pathology

The final pathology was a papillary tumor of the pineal region, WHO grade II. Patient did well postoperatively but developed vertical diplopia. Her extraocular muscles are intact. She had mild light-near dissociation, consistent with dorsal midbrain syndrome. On 1-month follow-up, she continued with vertical diplopia and significant amount of blinking. She was seen by neuro-ophthalmology and fitted for a pair of prism glasses. On 3-month follow-up, she was blinking less, and her vertical diplopia was significantly improved. She eventually was able to drive and return to work.

### 9:38 Postoperative Visit

At her 3-month postop visit, she noted less vertical diplopia and less issue with blinking. This is her 3-month postop MRI showing complete tumor resection and a functioning third ventriculostomy.

## Acknowledgments

We thank Christopher Brown for help editing the video.

## Author Contributions

Primary surgeon: Shah. Assistant surgeon: Huff. Editing and drafting the video and abstract: all authors. Critically revising the work: all authors. Reviewed submitted version of the work: Shah, Witten. Approved the final version of the work on behalf of all authors: Shah. Supervision: Shah.
